# Decoding metastatic microenvironments through single-cell omics reveals new insights into niche dynamics and tumor evolution

**DOI:** 10.1371/journal.pbio.3003299

**Published:** 2025-07-14

**Authors:** Fengshuo Liu, Xiang H.-F. Zhang

**Affiliations:** 1 Lester and Sue Smith Breast Center, Baylor College of Medicine, Houston, Texas, United States of America; 2 Dan L Duncan Cancer Center, Baylor College of Medicine, Houston, Texas, United States of America; 3 Graduate Program in Cancer and Cell Biology, Baylor College of Medicine, Houston, Texas, United States of America; 4 McNair Medical Institute, Baylor College of Medicine, Houston, Texas, United States of America; Princeton University, UNITED STATES OF AMERICA

## Abstract

Metastasis is the predominant cause of cancer mortality, primarily driven by complex tumor–host interactions within specialized metastatic niches. Recent advances in single-cell technologies have provided unprecedented insights into metastatic niche formation, evolution and function, including how primary tumors precondition distant organs for metastases and how disseminated tumor cells dynamically interact with host cells to modulate their environments. Integrated single-cell studies across multiple cancer types have also revealed divergent and convergent metastatic adaptation strategies. These findings collectively highlight metastasis as a dynamic, cooperative process shaped by intricate tumor–host interactions, and provide a foundation for novel therapeutic strategies targeting components of the metastatic microenvironment.

## Abstract 2

Recent advances in single-cell technologies have provided unprecedented insights into metastatic niche formation, evolution and function. This Essay explores the use of single-cell technologies, including integrative and spatial methods, in mapping and analyzing metastatic niches, and investigates how to harness insights gained to inform novel therapeutic interventions.

## Introduction

Metastasis is the leading cause of cancer-related mortality, accounting for the vast majority of cancer deaths [1,2]. The poor prognosis associated with metastasis arises from its complex, multi-step nature and the essential role played by the tumor microenvironment in supporting disseminated tumor cells (DTCs) in forming secondary lesions. This metastatic niche—initially proposed as a permissive tissue microenvironment in secondary organs—provides essential support for circulating tumor cells to survive, evade immune detection and proliferate into overt metastatic lesions [3–6].

Metastatic niches exhibit significant cellular and molecular heterogeneity, reflecting diverse and dynamic interactions among tumor cells and stromal cell populations. Traditional bulk tissue analyses have contributed valuable insights into metastatic niches, but lack the resolution to dissect their complexity, as bulk profiling averages signals from heterogeneous populations, obscuring key interactions and rare cell states. By contrast, single-cell technologies and other high-dimensional analyses (Box 1) enable researchers to identify unique cellular states, map intercellular communication networks, and trace the evolution of metastatic lesions at unprecedented resolution.

Box 1 **Advanced single-cell and high-dimensional technologies and analytic methods for metastasis research.**

**Single-cell RNA sequencing (scRNA-seq)**

scRNA-seq captures gene expression from individual cells, starting with single-cell isolation, mRNA conversion to cDNA, and sequencing. It enables the identification of diverse cell types, rare populations, and cellular states that are obscured by bulk analysis. Researchers use scRNA-seq to infer cell differentiation, immune responses, and cell–cell communication by analyzing ligand–receptor interactions [7].

**Single-cell ATAC sequencing (scATAC-seq)**

scATAC-seq measures chromatin accessibility in single cells using a Tn5 enzyme that tags accessible DNA regions with sequencing adapters. It enables the identification of active regulatory regions (promoters, enhancers, etc.) controlling gene expression in specific cells. Integrating scATAC-seq with scRNA-seq links gene expression to chromatin regulation, clarifying how regulatory elements drive cell type differences [8].

**Spatial transcriptomics**

Spatial transcriptomics measures gene expression directly within intact tissues, preserving spatial information lost in conventional scRNA-seq. Tissue sections are placed on slides coated with spatial barcodes that capture mRNA for sequencing, linking gene expression profiles to precise locations. This enables mapping of local molecular signals, cell distributions, and niche interactions essential for understanding tumor biology [9].

**Multimodal single-cell analysis**

Multimodal approaches combine multiple data types (e.g., transcription, chromatin accessibility, protein expression) from the same cells or samples. Examples include paired scRNA-seq and scATAC-seq or CITE-seq, which measure proteins alongside RNA. Such integration improves cell type definitions, reduces noise, and provides deeper insights into complex cellular states, which can be unclear when using a single method [10].

**Multiplexed imaging**

Multiplexed imaging spatially maps dozens of proteins within tissue sections. Co-detection by indexing (CODEX) employs iterative fluorescent labeling with DNA-tagged antibodies, enabling visualization of ~60 proteins per cell. Imaging mass cytometry labels proteins with metal-tagged antibodies, analyzed by mass spectrometry, achieving similar multiplexing. Both preserve tissue architecture, clarifying spatial interactions and cellular niches [11–13].

**Tumor niche labeling**

Both mCherry (fluorescent)-based and Sortase-A-based methods label tumor-adjacent cells (i.e., the niche). mCherry-based methods secrete fluorescent proteins, passively labeling nearby stromal and immune cells. By contrast, Sortase-A-based microenvironment niche tagging (SAMENT) uses the bacterial Sortase A enzyme to actively and covalently tag adjacent cells upon direct contact. While mCherry labeling is simpler, SAMENT provides higher specificity and control, ideal for precise niche cell identification and isolation [14,15].

**Lineage tracing**

Lineage tracing tags individual cancer cells with unique genetic barcodes, enabling researchers to track their descendants during tumor progression. Sequencing barcodes in tumors and metastases allows the identification of clonal relationships, clarifying how metastases originate, evolve, and spread. Barcoding helps answer the questions of whether metastases are monoclonal or polyclonal, their clonal origins and which subpopulations drive metastasis [16–18].

Single-cell sequencing has been instrumental in uncovering previously unrecognized cellular genomic and transcriptomic diversity within metastatic niches, revealing distinct gene expression programs in both tumor and stromal cell populations. Recent technological advances, including high-dimensional multiplexed imaging and spatial transcriptomics, enhance these insights by providing spatial context, capturing dynamic interactions between DTCs and surrounding host cells within the tissue microenvironment. Table 1 summarizes recent technological developments and their applications in metastasis research. Together, single-cell sequencing and spatial and multiplexed imaging allow direct visualization of molecular changes and functional behaviors at the single-cell level, and have profoundly enhanced our understanding of metastasis biology.

**Table 1:** Milestones in high-resolution/throughput technologies in metastasis research.

Year	Method/topic	Significant insight/advance
1972	Flow cytometry and FACS	FACS enables the isolation and analysis of cells of interest, facilitating early metastasis studies [19].
1996	Laser capture microdissection	Allows precise extraction of individual cells of interest from tissues for downstream analysis [20].
2002	Single-cell genomic hybridization	Analysis of DTCs reveals high genetic heterogeneity and post-dissemination selection [21].
2007	CTC capture chip	Microfluidic CTC-chip captures CTCs from patient blood, enabling real-time metastasis monitoring [22].
2008	Single-cell analysis of DTCs	HER2 amplification in metastasis-initiating cells is spotted, which had been missed by bulk tumor analysis [23].
2011	Single-cell genome sequencing of cancer	Reveals clonal evolution and selection of metastatic founder cells in breast cancer [24].
2014	CTC clusters drive metastasis	CTC clusters are highly metastatic, demonstrating that collective migration enhances metastasis [25].
2014–2015	Intra-tumoral transcriptomic heterogeneity	Studying glioblastoma tumors reveals coexisting molecular subtypes, emphasizing intra-tumoral heterogeneity [26].
	High-dimensional tissue imaging	IMC is introduced, allowing high-dimensional spatial analysis of metastatic TMEs [11].
	Droplet microfluidics in scRNA-seq	Droplet microfluidics scales up scRNA-seq, enabling the simultaneous profiling of thousands of tumor and immune cells [27].
2015–2016	scATAC-seq	scATAC-seq helps reveal clonal evolution and selection of metastatic founder cells in breast cancer [8].
	scRNA-seq in dissecting a metastatic melanoma ecosystem	scRNA-seq enables the identification of invasive and drug-resistant cell states within tumors [28].
2016–2017	Spatial transcriptomics	Spatial transcriptomics maps gene expression in situ, preserving the spatial context of metastatic niches [29].
	Single-cell multi-omics	CITE-seq integrates transcriptomics and surface protein profiling, improving metastatic cell classification [30].
2017–2018	Immune cells facilitating metastasis	CTC–neutrophil clusters are discovered, showing how immune cells aid CTC survival and enhance metastasis [31].
	Highly multiplexed immuno-imaging of tumors	CODEX and MIBI reveal structured TME within metastatic TNBC [12,13].
2019	Fluorescent-based metastatic niche labeling	Cancer-associated parenchymal cells exhibit stem-like traits and support tumor growth [15].
2020	Lineage tracing of metastasis	CRISPR-based lineage tracing reconstructs the evolutionary paths of metastatic clones in vivo [17].
2020–2025	Single-cell/special-resolved atlases of metastatic tumors	Single-cell atlases of metastatic cancers provide high-resolution maps of tumor heterogeneity and immune interactions [32–35].
2021	Lineage tracing and transcriptome recording	Metastatic pancreatic cancer cells arise from a rare hybrid EMT state, balancing growth and invasion [36].
2023	Spatial immune landscape of brain metastases	IMC analysis of brain metastases reveals distinct immune microenvironments compared to primary brain tumors [37].
2024	Multi-modal single-cell and spatial map of metastatic breast cancer	Multi-modal single-cell and spatial transcriptomics map metastatic breast cancer across multiple organ sites, identifying conserved metastatic cell states [38].
2024–2025	Fluorescent-based metastatic niche labeling in brain metastasis	TNBC forms perivascular sheaths, and HER2-positive breast cancer forms spheroids, evoking distinct microglial and stromal responses in brain metastasis [39].
	Sortase-A mediated niche labeling	SAMENT niche labeling revealed that ERα^+^ macrophages exclude T cells in metastatic niches, promoting cancer colonization across multiple organs [14].
	Fluorescent-based metastatic niche labeling in bone metastasis	Bone marrow macrophages essential for erythropoiesis are hijacked by metastatic tumor cells to acquire iron, impairing blood cell production, promoting cancer growth, and driving anemia in metastatic niches [40].

This table summarizes key technological advances that have deepened our understanding of tumor metastasis by enabling detailed investigation of cellular and molecular heterogeneity and interactions within metastatic environments. CITE-seq, cellular indexing of transcriptomes and epitopes by sequencing; CODEX, co-detection by indexing; CRISPR, clustered regularly interspaced short palindromic repeats; CTC, circulating tumor cell; DTC, disseminated tumor cell; EMT, epithelial–mesenchymal transition; ERα, estrogen receptor-α; FACS, fluorescence-activated cell sorting; HER2, human epidermal growth factor receptor 2; IMC, imaging mass cytometry; MIBI, multiplexed ion beam imaging; SAMENT, Sortase-A-based microenvironment niche tagging; scATAC-seq, single-cell assay for transposase-accessible chromatin with high-throughput sequencing; scRNA-seq, single-cell RNA sequencing; TNBC, triple negative breast cancer; TME, tumor microenvironment.

Advanced high-resolution omics analyses and models have increasingly been employed as discovery approaches to initiate investigations, validate methods to translate findings from animal models to human patients, or to conduct detailed mechanistic studies in animal systems informed by clinical observations. Specifically, single-cell analyses have been critical in resolving cellular heterogeneity, identifying rare but biologically significant subpopulations and elucidating precise cell-state transitions typically obscured in bulk sequencing approaches. These technologies have enabled detailed mapping of cell–cell interactions, delineation of distinct immune, stromal and tumor cellular subsets, and revealed how these interactions establish immunosuppressive niches, drive metabolic adaptations, and promote metastatic progression. Studies at single-cell resolution have also uncovered subtle molecular programs and ligand–receptor interactions, providing essential insights for therapeutic targeting (Table 1).

In this Essay, we highlight key discoveries that have characterized the metastatic niche’s cellular and molecular landscape, explore the applications of cutting-edge single-cell technologies in mapping and analyzing metastatic niches and discuss the limitations of these approaches. We also consider future developments and emerging strategies to further dissect the metastatic niche, including integrative and spatial methods, and how to harness these insights to inform novel therapeutic interventions.

## Adaptive strategies and microenvironmental cross-talk in metastatic colonization

Successful metastatic colonization requires tumor cells to deploy adaptive strategies that enable them to survive and proliferate within distinct microenvironments at distant sites. In this section, we outline key mechanisms underlying metastatic adaptation, focusing on how tumor cells communicate with host tissues to establish pre-metastatic niches, regulate dormancy, remodel immune responses, and undergo metabolic reprogramming.

### Micrometastases and pre-metastatic niche formation

**Fig 1:**
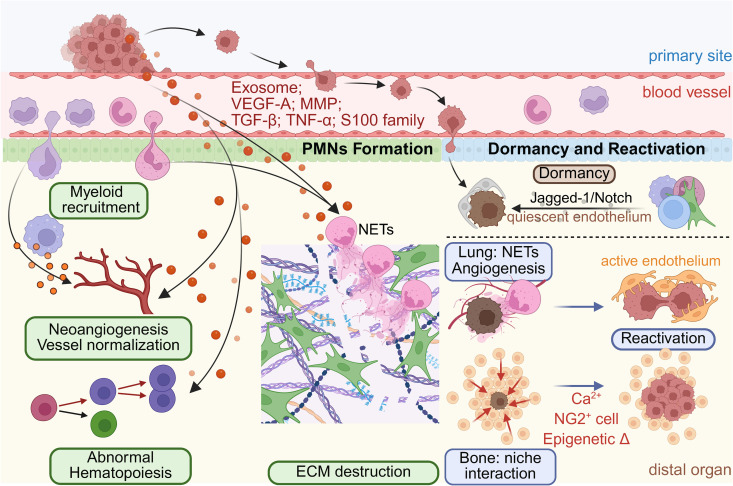
Single-cell insights into pre-metastatic niche formation, tumor dormancy, and reactivation dynamics. Schematic representation illustrating critical events in metastatic niche formation and progression revealed by single-cell analyses. Primary tumors initiate the creation of pre-metastatic niches (PMNs) through the release of tumor-derived exosomes and factors, which recruit myeloid-derived cells and induce stromal remodeling in distant tissues, establishing an immunosuppressive and fibrotic microenvironment prior to tumor cell arrival. Upon arrival, disseminated tumor cells may enter dormancy, regulated by niche interactions involving immune and vascular cells. Dormant cells are maintained by interactions with stable vasculature, immune surveillance, and signaling pathways such as Jagged-1/Notch, but can reactivate when interacting with niche cells. ECM, extracellular matrix; MMP, matrix metalloproteinase; NET, neutrophil extracellular trap.

Before metastases become clinically apparent, primary tumors alter distant microenvironments, creating specialized ‘pre-metastatic niches’ that support DTCs (Fig 1). Early studies identified bone marrow-derived myeloid cells infiltrating future metastatic sites, secreting factors that remodel local vasculature and extracellular matrix (ECM) [6]. Tumor-derived exosomes contribute to niche formation by inducing pre-metastatic changes in distant tissues [41]. These findings intriguingly demonstrate the systemic communication capabilities of tumors via exosomes, underscoring their potential as therapeutic targets or biomarkers for early metastatic detection. Additionally, tumor-secreted factors such as lysyl oxidase (LOX) initiate ECM remodeling via collagen crosslinking and monocyte recruitment, enhancing niche readiness [42]. LOX-driven ECM remodeling is also a potential therapeutic target, given its essential role in physically reshaping tissues to accommodate tumor growth. VEGFR1-expressing myeloid cells from bone marrow also play crucial roles, underscoring the complexity of cellular interactions during metastatic niche formation [43]. Identifying these VEGFR1^+^ cells suggests possible utility in targeting this cell population specifically to prevent or limit metastasis.

**Fig 2:**
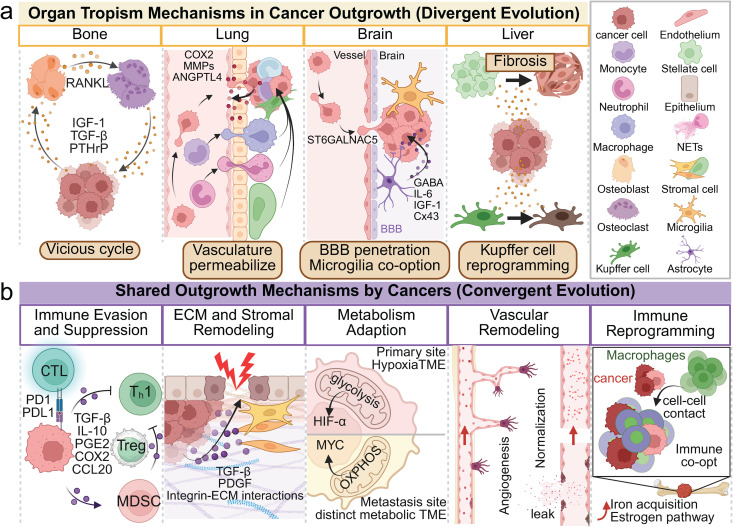
Divergent and convergent mechanisms in metastatic colonization. **A.** Tissue-specific mechanisms underlying metastatic colonization. Disseminated tumor cells (DTCs) engage in distinct interactions with organ-resident cells upon arrival at distant sites. Cancer cells adaptively reprogram the local environment to facilitate survival and expansion. For example, in bone, tumor-secreted cytokines disrupt osteoblast–osteoclast homeostasis, promoting a cycle of bone remodeling and tumor growth. Such adaptations depend on both tissue-specific features and intrinsic tumor properties. **B.** Shared mechanisms facilitating metastatic colonization. DTCs commonly employ strategies to evade immune detection, including recruitment of immunosuppressive cell populations, suppression of natural killer and T cell functions, and intrinsic metabolic reprogramming. These shared mechanisms are broadly conserved across diverse cancer types and metastatic sites. BBB, blood–brain barrier; CTL, cytotoxic T lymphocyte; ECM, extracellular matrix; MDSC, myeloid-derived suppressor cell; MMP, matrix metalloproteinase; NET, neutrophil extracellular trap; OXPHOS, oxidative phosphorylation; T_H_1, T helper 1 cell; TME, tumor microenvironment; Treg, regulatory T cell.

The advent of single-cell analyses has significantly refined our understanding of pre-metastatic niches, uncovering previously unrecognized cellular heterogeneity and molecular interactions at single-cell resolution. For example, single-cell RNA sequencing (scRNA-seq) of pre-metastatic lung microenvironments revealed expansions of pro-inflammatory neutrophils that orchestrate early niche formation, remodeling the ECM and promoting the outgrowth of dormant DTCs [44] (Fig 2A). A recent study using scRNA-seq uncovered a previously unknown tumor-induced interaction between osteoprogenitor (OP) cells and granulocyte-monocyte progenitor (GMP) cells within the bone marrow ecosystem [45]. Extracellular vesicles containing HTRA1 drive OPs to overexpress matrix metalloproteinase 13, promoting GMP expansion and systemic immunosuppression. Single-cell approaches were crucial in these studies for identifying specific cellular populations and their molecular interactions, demonstrating a complex, systemic remodeling initiated long before overt metastasis. Critically, this abnormal tumor-induced myelopoiesis and the resulting immunosuppressive environment are therapeutically targetable through IL-1β inhibition, which effectively normalized myeloid differentiation and significantly reduced metastatic spread [45,46]. These findings underscore the importance of the tumor-driven microenvironmental alterations preceding overt metastatic colonization.

### Dormancy and reactivation of disseminated cells

DTCs frequently enter a dormant state within distant organs, surviving as microscopic lesions for prolonged periods before reactivating into overt metastases. A study that used 3D co-culture models and intravital imaging demonstrated that DTCs remain in a growth-arrested state when positioned on stable capillaries but transition to active proliferation upon association with angiogenic vessels [47]. Thus, the condition of the local vasculature has emerged as a critical regulator of tumor dormancy. Conversely, distinct niche-derived signals can initiate the reactivation of dormant cells. Several seminal single-cell-informed studies have neutrophils and neutrophil extracellular traps (NETs) as essential triggers for DTC reactivation. Specifically, NETs deposit proteases and pro-inflammatory mediators, degrade dormancy-maintaining ECM components and reactivate dormant tumor cells [48] (Fig 1). Clinically, these findings align with observations linking pro-inflammatory responses or tissue injury to subsequent metastatic flare-ups [49,50].

Various molecular mechanisms underlying DTC dormancy and reactivation in bone have been explored using high-resolution imaging combined with mouse models. Bone metastasis initiation closely couples with bone remodeling processes, particularly involving NG2^+^ mesenchymal stem/stromal cells. NG2^+^ cells promote cancer cell proliferation and migration through direct cell–cell interactions mediated by N-cadherin [51]. Importantly, osteogenic niches within the bone microenvironment transiently reduce estrogen receptor (ER) expression in micrometastases of ER^+^ breast cancer cells via epigenomic reprogramming mediated by EZH2, facilitating endocrine resistance and enhanced cellular plasticity, which can potentially reactivate the dormant cancer cells [52]. Furthermore, heterotypic adherens junctions formed between cancer and osteogenic cells activate mTOR signaling, which is crucial for progressing from micrometastases to macrometastases [53]. Lastly, osteogenic cells act as calcium reservoirs, directly transferring calcium to cancer cells through gap junctions, thereby boosting intracellular calcium signaling, further promoting dormant metastatic tumor exit latency and aggressive progression [54].

Dormancy is increasingly being recognized not as a passive state, but rather as an actively maintained balance modulated by precise niche-derived signals, including local vasculature stability, NET production, NG2^+^ mesenchymal stem/stromal cell interactions, osteogenic niche-driven epigenomic reprogramming, and calcium signaling through heterotypic cell junctions. Disruption of this equilibrium by pro-inflammatory, stromal, or cellular cues prompts metastatic reactivation. Understanding these intricate cellular and molecular interactions opens promising therapeutic avenues to maintain dormancy, inhibit reactivation, and ultimately prevent metastatic progression.

### Metabolic adaptations in metastatic outgrowth

Colonization of distant organs requires significant metabolic flexibility from DTCs. Metastatic cells commonly reprogram their metabolic activities to accommodate distinct nutrient availability and oxygenation status at new metastatic sites; for instance, metastatic tumor cells frequently shift towards oxidative metabolism. Analysis of breast cancer patient-derived xenograft models has demonstrated that primary tumor cells predominantly activate hypoxia and glycolysis pathways, while their corresponding lung metastases exhibit enrichment in oxidative phosphorylation and MYC-regulated metabolic programs [55] (Fig 2B). A compelling illustration of organ-specific metabolic adaptation arises from brain metastases. Breast cancer cells metastasizing to the brain adopt neuronal metabolic characteristics, specifically utilizing the neurotransmitter gamma-aminobutyric acid (GABA) as an energy source. High-resolution imaging with biochemical assays have revealed that brain-tropic breast cancer cells significantly overexpress GABA transporters and related metabolic enzymes, facilitating GABA import from the neural microenvironment and subsequent catabolism into succinate and NADH for energy production [56]. This specialized metabolic strategy, involving the exploitation of a brain-specific metabolite, exemplifies how metastatic cells adapt metabolically to the organ-specific microenvironment. These findings collectively emphasize that metastatic tumor cells undergo metabolic reprogramming—exploiting niche-specific nutrients such as neural-derived GABA to enhancing antioxidant production—to support survival and proliferation in distal tissue contexts.

### Immune remodeling by metastatic cells

The ability to evade or suppress immune responses is a hallmark of successful metastatic progression. Single-cell resolution studies have revealed that metastatic sites frequently develop specialized immune microenvironments that favor tumor cell survival. Although immune surveillance can eliminate numerous DTCs, those cells that successfully form macrometastases typically do so within immunosuppressive niches. Such environments often exhibit dysfunctional or excluded cytotoxic immune cell activity, accompanied by an increased presence of regulatory and pro-tumoral immune populations.

Single-cell and spatial transcriptomic analyses of matched primary and metastatic colorectal cancer samples have uncovered an immunosuppressive niche in liver metastases, characterized by heterogeneous populations of macrophages and neutrophils. These analyses identified a distinct, metabolically active pro-tumoral macrophage subtype (MRC1^+^CCL18^+^) dominating the metastatic niche [57]. Another study identified osteopontin (SPP1)^+^ ‘foam cell’ macrophages and reactive fibroblasts that promoted immune suppression by enriching regulatory T cells and depleting cytotoxic T cells in colorectal liver metastases [58]. In breast and lung cancer brain metastases, resident microglia and macrophages adopt wound-healing states, resulting in excessive collagen deposition by reactive astrocytes and fibroblasts and transforming the normally ECM-poor brain tissue into a fibrotic, tumor-supportive environment [59] (Fig 2B). Lung cancer bone metastases create senescent, pro-angiogenic niches involving senescent-like tumor cells, stressed CD4^+^ T cells, and endothelial-to-mesenchymal transitions. Here, SPP1 facilitates communication among tumor cells, T cells, and endothelial cells, enhancing angiogenesis and immune suppression via endothelial SOX18 activation [60]. Similarly, prostate cancer bone metastases establish an immunosuppressive bone marrow environment with exhausted T cells and specialized macrophages driven by the CCL20–CCR6 chemokine axis; targeting this pathway restored immune responses and inhibited tumor growth in mouse models [61].

Single-cell studies that focus on hematopoietic stem and progenitor cell subtypes have revealed that metastatic tumors rich in IL-6 alter bone marrow hematopoiesis, favoring immunosuppressive myeloid cell production at the expense of other lineages, creating a systemic pro-metastatic immune environment [62]. Furthermore, tumor-bearing mice exhibit inflamed endothelial cells, hypothalamic inflammation, and elevated appetite-suppressing factors such as lipocalin-2, linking metastatic tumors directly to altered central metabolism and appetite regulation [63]. Thus, metastases profoundly impact systemic physiology beyond local niches.

### Stromal cell contributions to metastatic outgrowth

In addition to immune cells, various stromal cells—including fibroblasts, endothelial cells, pericytes, and astrocytes—are co-opted by metastatic tumors to establish a supportive niche. A population of cancer-associated parenchymal cells (CAPs) were discovered in breast cancer lung metastases using a fluorescent niche labeling approach and scRNA-seq [15]. The CAPs originated from normal lung alveolar epithelial cells that acquired stem-like and mesenchymal characteristics under the influence of nearby tumor cells. These reprogrammed epithelial cells produced factors that supported metastatic growth, demonstrating the hijacking of non-immune stromal cells to favor colonization. Similarly, in brain metastases, resident astrocytes become key niche players, forming gap junctions with tumor cells and secreting molecules (e.g., cytokines such as IL-6 and IGF-1) that enhance cancer cell survival and blunt T-cell attacks [64–66] (Fig 2A). Inhibition of the gap junction protein connexin 43, essential for astrocyte–tumor interactions, can effectively suppress metastatic growth in mouse models [67,68] suggesting that targeting astrocytes and their gap junctions with tumor cells could have therapeutic potential in treating brain metastasis.

Endothelial cells can also significantly influence metastatic niches, depending on their activation state. As previously noted in the dormancy context, endothelial cells in a quiescent state maintain tumor dormancy, whereas activated, angiogenic endothelial cells can stimulate metastatic cell proliferation [47]. New vasculature in metastases often expresses unique adhesion molecules, facilitating further cancer cell recruitment and immune cell trafficking. Furthermore, perivascular cells such as pericytes secrete chemokines that result in tumor cell attraction [64–66] (Fig 2B). The results of these single-cell investigations collectively portray metastasis as a collaborative effort wherein metastatic tumor cells induce reprogramming of surrounding stromal cells, converting them into supportive niches. Each stromal cell type, whether fibroblast, epithelial cell, or astrocyte, is actively reshaped by metastatic tumor cells, reinforcing the concept of metastasis as a complex, cooperative process where local tissue components are remodeled to facilitate tumor growth.

## Clonal heterogeneity underlies convergent and divergent metastatic patterns

Metastatic cancer cells encounter diverse ‘seed and soil’ conditions across various organs. Although only a small subset of circulating tumor cells colonizes distant sites successfully, those that do must adapt specifically to each organ’s unique microenvironment or niche. In this section, we first explore recent single-cell studies on organ tropism by using breast cancer as a primary example, detailing how distinct distal tissues (‘soil’) employ unique mechanisms to support DTCs. Next, we discuss the clonality of metastatic tumor cells (‘seeds’) from the viewpoint of polyclonal metastasis. Last, we highlight recent integrated single-cell sequencing analyses, illustrating divergent and convergent colonization strategies utilized by different cancers at common metastatic sites.

### Organ tropism in metastasis

Breast cancer frequently metastasizes to bone, lung, liver, and brain, each providing specialized niches that tumor cells utilize during colonization (Fig 2A). In bone, breast cancer cells activate osteoclast-mediated bone resorption through the RANKL–RANK or TGF-β–PTHrP-driven cycle, releasing growth factors that further promote tumor growth [69,70]. This niche is characterized by fibrosis, immune suppression, and an abundance of cancer-associated fibroblasts (CAFs) [71]. The liver presents another challenge, as resident Kupffer cells rapidly clear circulating tumor cells [72]. Breast cancer-derived integrin-rich exosomes contribute to hepatic niche formation, suppressing immune responses [73] and inducing hepatic stellate cells to differentiate into protective CAFs, creating a fibrotic and immunosuppressive environment [74]. In the lung, breast cancer cells must evade innate immune defenses such as natural killer cells and neutrophils [75,76]. Such immune evasion is mediated by tumor cells releasing pro-inflammatory and proteolytic factors, including COX2, MMPs, and angiopoietin-like 4, to enhance vascular permeability and extravasation [77–80]. Brain metastasis involves overcoming the blood–brain barrier (BBB), often facilitated by tumor-specific molecules such as alpha2,6-sialyltransferase [81]. Upon successful colonization, metastatic cells form gap junctions with astrocytes, altering astrocyte–immune signaling pathways to foster tumor growth and resistance to apoptosis. Additionally, microglia adopt immunosuppressive roles, while adaptive immune infiltration remains limited due to the BBB [82–85].

These distinct niches in bone, liver, lung, and brain collectively exemplify how cancer cells exploit organ-specific strategies through tailored molecular adaptations, stromal cell engagement, and immune modulation. Researchers have employed rigorous experimental approaches to uncover these niche-specific mechanisms and to identify previously unrecognized cellular subsets: sampling metastatic tissues from individual organs, profiling them using advanced single-cell sequencing and spatial imaging technologies, and performing comparative transcriptomic analyses. However, a notable gap remains in understanding the dynamic interplay between these diverse niches and how metastatic cells simultaneously adapt their strategies across multiple organ microenvironments. Future research should integrate multi-organ sampling and longitudinal single-cell analyses to unravel how tumor cells dynamically coordinate these complex interactions over time. Such integrative studies hold significant promise for designing therapies targeting universal and niche-specific vulnerabilities, ultimately improving outcomes for patients with metastatic lesions.

### Polyclonal heterogeneity in metastasis

The ‘seed and soil’ hypothesis by Stephen Paget originally proposed that successful metastatic colonization occurs only when tumor cells (‘seeds’) encounter an organ microenvironment (‘soil’) conducive to their growth [86]. Modern genomic and lineage-tracing studies have expanded this theory, revealing that metastases frequently arise through polyclonal seeding, in which genetically heterogeneous tumor cell clusters jointly colonize distant sites, significantly enhancing metastatic efficiency compared to single-cell dissemination [87] (Table 1). Such cooperative colonization highlights that metastasis success depends equally on the microenvironmental conditions of the target organ and the intrinsic, collective properties of the DTCs.

Single-cell analyses have illuminated the role of tumor cell clonality and organ-specific colonization mechanisms in metastasis. Polyclonal heterogeneity has been found in gastric cancer metastases across multiple organs, revealing specific tumor subpopulations adapted to tissue-specific niches and underscoring the role of tumor heterogeneity in metastatic specificity [88]. Similarly, scRNA-seq and spatial transcriptomics have been used to show that colorectal cancer metastases to the liver and ovary involve distinct tumor subclusters with unique molecular interactions tailored to the respective organ microenvironments; tumor cells exhibiting DLL4–NOTCH signaling were associated with ovarian colonization, whereas tumor cells with similar expression patterns to cholangiocytes were associated with liver metastasis [89]. These studies integrate and expand upon Paget’s original ‘seed and soil’ theory, illustrating that polyclonal dynamics and specific cellular adaptations critically shape metastatic outcomes across diverse organs.

### Convergent and divergent metastasis

We believe that the concepts of tumor polyclonal metastasis and convergent–divergent adaptation are closely interconnected, representing complementary aspects of metastatic complexity. Polyclonal seeding implies that heterogeneous tumor cell populations cooperatively colonize distant organs, potentially enhancing metastatic success through diverse yet collaborative biological strategies. This intra-tumoral diversity enables metastatic clusters to flexibly exploit multiple adaptive pathways simultaneously, setting the stage for both convergent mechanisms (shared adaptations across different tumors colonizing the same organ) and divergent strategies (reflecting specific traits shaped by their tissue of origin). Thus, polyclonal dynamics serve as a foundation for understanding how tumor cells achieve metastatic efficiency by simultaneously leveraging common niche-specific pathways and uniquely tailored adaptations.

Recent integrated single-cell analyses across different cancers colonizing the same organ have further revealed convergent and divergent adaptation strategies. A comprehensive transcriptomic meta-analysis across multiple cancer types revealed organ-specific metastatic gene expression programs that are consistently activated by diverse tumors metastasizing to the same site, such as neural signaling in brain metastases and hypoxia pathways in liver metastases [90]. Similarly, a direct comparison of brain metastases from lung adenocarcinoma and breast carcinoma at single-cell resolution revealed distinct tumor cell characteristics influenced by their tissues of origin; lung-derived tumor cells preserved alveolar lineage signatures, whereas breast-derived cells exhibited proliferative epithelial traits and stromal interactions [84]. Moreover, shared and distinct adaptation strategies have been noted among bone metastases from multiple primary tumor types; different cancers frequently converge on similar metastatic microenvironments, such as niches characterized by osteoclast-mediated bone resorption. Conversely, individual cancer types (e.g., lung cancers) can diverge by forming distinct immune archetypes, ranging from osteoclast-mediated niches to environments enriched with immunosuppressive CD4^+^ regulatory T cells and exhausted CD8^+^ T cells, underscoring the complex interplay between tumor origin and immune microenvironment adaptations [35]. Niche-labeling and single-cell analyses have further clarified convergent mechanisms used by diverse cancers metastasizing to bone. By using a Sortase A-based metastatic niche-labeling method (Box 1), ERα^+^ macrophages were identified that were specifically enriched in bone metastatic niches across multiple cancer types. Preprinted results suggest that these macrophages inhibited T cell infiltration via ERα signaling, promoting immune evasion and metastatic colonization [14]. Preprinted results of another study that used fluorescence-based niche labeling integrated with single-cell investigations revealed that metastatic tumors exploit iron-regulating macrophages in bone marrow, diverting iron metabolism from erythropoiesis toward tumor growth and leading to anemia [40]. The convergence of this iron-metabolic pathway across diverse cancer metastases highlights its potential as a common therapeutic target. Without such single-cell resolution and novel niche-labeling techniques, these intricate cellular adaptations and interactions would likely have remained undiscovered. These studies have shown that different cancers colonize the same distant organ through shared molecular pathways and unique tissue-of-origin-specific adaptations.

## Knowledge gaps and roadmap for future studies

A significant challenge in metastasis research is the technological limitation in reliably detecting microscopic metastatic niches, both clinically and preclinically. Traditional clinical imaging modalities, including MRI, CT, and PET, struggle to identify metastatic lesions smaller than approximately 1 cm^3^, resulting in microscopic niches remaining undetectable until substantial growth has occurred [91]. Similarly, it can be challenging to detect microscopic lesions in animal models without using invasive or terminal methodologies, complicating longitudinal studies on early metastatic events [92,93]. Consequently, early metastatic dissemination remains difficult to study due to intrinsic sensitivity limitations and the stochastic nature of metastatic spread.

Identifying rare cell populations, essential for understanding the initiation and progression of metastasis, is also significantly constrained by current technologies. Marker-based approaches such as fluorescence-activated cell sorting, imaging mass cytometry, and scRNA-seq depend on the use of predefined markers, meaning that rare or previously unidentified cell types can potentially be overlooked [94]. Additionally, single-cell transcriptomic data often display considerable sparsity and technical noise, obscuring subtle yet biologically relevant differences and possibly masking rare cells or unique functional states [95]. Although computational methods to overcome these limitations are continuing to evolve, significant improvements are still needed to resolve these technological constraints effectively [96].

Spatial and temporal limitations further hinder single-cell metastatic research. Current spatial transcriptomics technologies force trade-offs between resolution and transcriptomic depth; higher spatial resolution typically limits gene detection, whereas methods capturing comprehensive transcriptomic profiles lack actual single-cell resolution [97]. Additionally, the high cost and technical complexity of spatial technologies restrict their widespread adoption and hinder their application in longitudinal studies, leaving gaps in our understanding of time-resolved metastatic dynamics at the single-cell level [98]. There is thus a need for cost-effective, high-throughput spatial methodologies suitable for frequent longitudinal sampling to capture metastatic progression comprehensively.

Integrating multi-omics data also poses significant hurdles, primarily related to data sparsity, batch effects, and the risk of losing genuine biological variability during computational integration [99,100]. Effective integration of diverse data modalities, such as genomics, transcriptomics, epigenomics, and proteomics, demands sophisticated computational frameworks that preserve biological authenticity across heterogeneous datasets. Similarly, machine learning and artificial intelligence (AI) applications in single-cell analyses face constraints due to the requirement for large, accurately annotated datasets. The scarcity of high-quality annotated datasets often leads to biases or inaccuracies, particularly when distinguishing rare or previously uncharacterized cell states [96,101]. Enhancing the interpretability and biological relevance of AI models will be essential to maximize their utility in metastasis research.

Another issue to contend with is variability in tissue dissociation and harvesting protocols across laboratories, which can introduce batch effects that particularly impact complex tissues such as bone, which contains mineralized structures, or fatty tissues like the brain. These procedural differences may cause biased cell sampling and potentially mislead biological interpretations. Additionally, single-nucleus RNA sequencing, often used due to its practicality, captures only nuclear transcripts rather than comprehensive cellular RNA profiles, limiting data completeness. Multiplexed imaging approaches such as CODEX (Box 1) involve repeated probe hybridization and wash cycles, potentially weakening or distorting cell-surface marker signals. Furthermore, inconsistent data-processing standards can introduce analytical bias, complicating reproducibility across studies [102].

Addressing these limitations will necessitate innovative animal models and research methodologies explicitly designed for high-resolution metastasis studies. Engineered metastatic niches that attract DTCs into defined, accessible locations can facilitate the detection and analysis of early metastatic events without relying on invasive methods [36,91,103]. Additionally, niche-labeling techniques, in which tumor cells label adjacent niche cells, provide valuable spatial information and enable single-cell profiling independent of traditional spatial transcriptomics approaches [14,15,104]. Furthermore, advanced lineage-tracing methods utilizing CRISPR-based barcoding offer promising opportunities to dissect metastatic progression with unprecedented temporal resolution at the single-cell level [36,105,106].

These technological advances aim to help researchers to develop personalized metastasis prevention and treatment approaches. Utilizing high-resolution single-cell and spatial insights could allow for earlier and more accurate identification of patient-specific metastatic risks and therapeutic vulnerabilities [94]. For example, analyzing circulating tumor cells and profiling metastatic niches could predict the onset of metastasis, enabling tailored preventive measures or therapies targeting specific metastatic cell populations [107]. Incorporating these advanced methodologies into clinical practice represents a promising future direction toward personalized oncology, potentially transforming clinical outcomes through precisely timed and targeted interventions.

## Conclusions

Single-cell omics technologies have fundamentally transformed our understanding of metastatic niches, providing unprecedented insights into the complex cellular ecosystems underpinning cancer metastasis. By dissecting cellular heterogeneity at high resolution, these advanced methodologies have revealed the dynamic evolution of metastases through interactions between DTCs and diverse host cell populations. Techniques such as scRNA-seq and advanced imaging have enabled the identification of distinct cellular phenotypes and molecular states that characterize metastatic niches, highlighting that pre-metastatic niche formation, dormancy, immune evasion, and metabolic adaptation are actively orchestrated processes driven by intricate cellular communication within the microenvironment.

Despite remarkable progress, significant challenges persist. Current technologies often lack the sensitivity to detect early metastatic dissemination, hindering longitudinal studies focused on initial niche establishment and subsequent progression. Additionally, existing single-cell approaches encounter limitations due to dependence on predefined markers, data sparsity, technical noise, and restricted spatial-temporal resolution. Addressing these issues will necessitate novel multidisciplinary approaches integrating advanced spatial transcriptomics, lineage-tracing methodologies, and multiomics analyses enhanced by sophisticated computational frameworks and AI.

Looking forward, advancements in these technological domains will unlock unprecedented opportunities to investigate metastasis. These innovations will facilitate detailed mapping of niche dynamics across time, uncovering organ-specific and common metastatic adaptation mechanisms shared among various cancers. Clinically, such discoveries could enable personalized strategies for metastasis prevention and targeted treatment by revealing critical vulnerabilities within metastatic cells and their niches. Single-cell techniques thus hold promise in bridging fundamental metastasis research with clinical oncology applications, paving the way for precise targeting of metastatic disease. By elucidating the complexities of metastatic niches at single-cell resolution, researchers are positioned to revolutionize metastasis prevention and treatment, significantly enhancing cancer prognosis and transforming patient care.

## Abbreviations

AI: artificial intelligence
BBB: blood–brain barrier
CAPs: cancer-associated parenchymal cells
CODEX: co-detection by indexing
DTCs: disseminated tumor cells
ECM: extracellular matrix
ER: estrogen receptor
GABA: gamma-aminobutyric acid
GMP: granulocyte-monocyte progenitor
LOX: lysyl oxidase
NETs: neutrophil extracellular traps
OP: osteoprogenitor
SAMENT: Sortase-A-based microenvironment niche tagging
